# Traumatic pseudoaneurysm in brachial artery after removal of a subdermal contraceptive implant

**DOI:** 10.1590/1677-5449.200040

**Published:** 2020-09-21

**Authors:** Rafael Borges Monteiro, Patrick Bastos Metzger, Ariadne Bonachela de Moura, Antonio Herculano Silva, Matheus Nogueira Campos, Arthur Suana de Brito, Mirella Prado Luengo, Maria Júlia Andrade Nascimento

**Affiliations:** 1 Hospital Municipal do Campo Limpo – HMCL, Departamento de Cirurgia Vascular e Endovascular, São Paulo, SP, Brasil.; 2 Hospital Universitário Professor Edgard Santos, Salvador, BA, Brasil.; 3 Centro Universitário das Américas – CAM, Curso de Medicina, São Paulo, SP, Brasil.; 4 Beneficência Portuguesa de São Paulo, Departamento de Cirurgia Vascular, São Paulo, SP, Brasil.; 5 Universidade Anhembi Morumbi – UAM, São Paulo, SP, Brasil.

**Keywords:** vascular injury, brachial artery, contraceptives

## Abstract

The frequency of invasive therapeutic procedures has increased as medicine evolves, and the number of complications related to them has increased as a consequence. Subdermal contraceptive implants (SCI) offer benefits for female contraception, but implant and removal are associated with a complication rate of around 3%. In this article, we report and discuss a case of traumatic brachial artery pseudoaneurysm after an attempt to remove an SCI, complicated by compression of the median nerve.

## INTRODUCTION

Pseudoaneurysms are an increasingly common complication, because of the significant increase in invasive, diagnostic, and therapeutic procedures.^1^^,^^2^ Brachial artery pseudoaneurysm is a rare condition that occurs in fewer than 0.04% of cases.^1^ The most common etiology is iatrogenic (puncture or diagnostic tests and examinations). It occurs in 0.3 to 0.7% of patients and causes increased morbidity and mortality, longer hospital stays, and higher healthcare expenditure.^1^^,^^2^

The frequency of subdermal contraceptive implant (SCI) use for contraception and hormone replacement has been increasing over the last 5 years and is associated with a total complication rate of around 3%.^3^ Here, the authors present an uncommon clinical case of a brachial pseudoaneurysm caused by attempted removal of an SCI and complicated by compression of the median nerve.

## CASE DESCRIPTION

The patient was an 18-year-old, primiparous woman who was admitted to hospital by the gynecology and obstetrics service for removal of an SCI that had been implanted 1 month previously. The implant had been placed in the medial arm, but after the procedure the patient had suffered from pain, localized swelling, and ecchymosis. At an outpatients follow-up consultation to review the case, the patient exhibited paresthesia, progressive loss of finger flexion strength, and hypoesthesia in the field of the left median nerve. The gynecology team therefore decided to remove the device prematurely during the postoperative outpatients follow-up visit.

**Figure 1:**
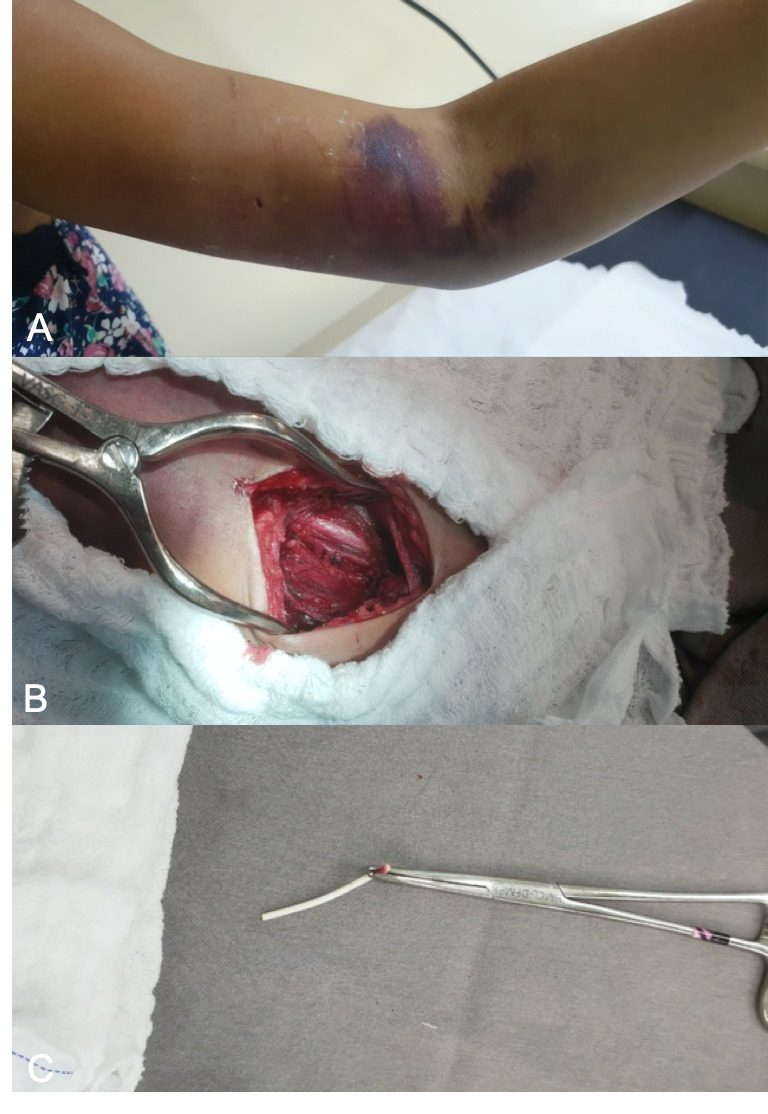
(**A**) Large-volume hematoma in the distal third of the left arm. Note the medial incision in the mid third of the arm, made for the attempt to remove the subdermal contraceptive implant; (**B**) Open surgery to repair the brachial pseudoaneurysm. Complete removal of the hematoma, decompression of the nerve, and repair of the orifice in the artery; (**C**) Contraceptive implant after removal.

**Figure 2:**
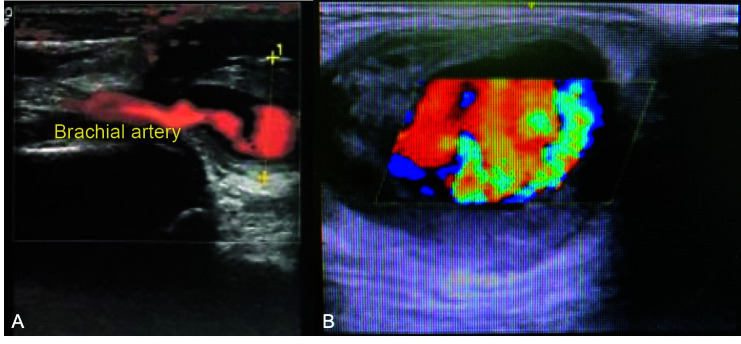
(**A**) Preoperative longitudinal Duplex ultrasonography showing where the pseudoaneurysm interrupts the artery; (**B**) Turbulent “yin yang” flow in the pseudoaneurysm.

The gynecology team reported that during initial surgical manipulation they found removal of the device to be extremely difficult, using a surgical approach via the medial aspect of the arm at the proximal extremity of the implant, and decided to abort the procedure. Afterwards, the patient experienced significant localized pain and was unable to fully extend her arm, which was swollen. A pulsating mass was observed, and distal paresthesia was detected. At this point, on the same day as the attempted removal, the vascular surgery team was called in, because of a suspected brachial pseudoaneurysm (Figure 1). Physical examination revealed a pulsating mass in the medial aspect of the distal third of the patient’s arm. The SCI could not be located by palpation, probably because of the volume of local hematoma. Color Doppler ultrasound showed a pseudoaneurysm in the medial aspect of the brachial artery, with thrombi in the wall, diameters of 4.2 cm x 3.5 cm, a 6 mm neck, and turbulent flow (Figure 2).

Immediate open surgical intervention was initiated to repair the injury, in view of the neurological involvement. The procedure was performed under local anesthesia and mild sedation, via a medial incision in the arm. The injury to the brachial artery was identified and local compression of the nerve was observed. The arterial orifice was sutured and the local hematoma was evacuated. In immediate postoperative recovery, the patient presented strong and symmetrical distal pulses, adequate perfusion, and unimpaired distal motricity and sensitivity.

During follow-up, the patient recovered within 14 days with total remission of pain and recovery of motor capacity, full extension of the forearm, and no flexion deficit, paresthesia, or hypoesthesia of the fingers. Distal pulses remained strong and symmetrical bilaterally.

## DISCUSSION

A brachial artery pseudoaneurysm is a rare complication that is generally of traumatic or iatrogenic origin.^1^ It is most often seen in injecting drug users and hemodialysis patients.^1^^,^^2^ In this paper, we describe an uncommon case of iatrogenic brachial pseudoaneurysm caused when attempting to remove an SCI. These complications can progress to conditions such as hemorrhagic rupture, compression of nerves and veins, formation of significant edema that restricts distal perfusion, and localized necrosis of the skin because of ischemia.^1^^,^^2^^,^^4^^,^^5^

Pseudoaneurysms measuring 2 cm or less tend to be asymptomatic. The majority of symptomatic cases present with pulsating masses. If sepsis is present, it may be associated with brachial pseudoaneurysms involving infection, caused by chronic injected drug use.^1^ Risk factors for pseudoaneurysm formation include diabetes mellitus and platelet counts below 200,000/ µL. Use of preoperative or postoperative anticoagulants and/or platelet antiaggregants is also a risk factor. Other factors include advanced age, female sex, and elevated body mass index (BMI).^2^^,^^4^^,^^5^

Implanting or explanting subdermal contraceptive devices can increase the frequency of vascular and neurological injuries.^3^ Insertion and later removal of these implants is within the expertise expected of a specialist.^3^ The majority of health services require training, provided by the SCI manufacturer, before physicians manage implants and explants. Failure to follow the implant instructions can result in deep implantation that cannot be palpated, involving risk of implant migration, neurological and vascular injuries, and, rarely, intravascular implantation.^3^ An SCI should be removed or substituted after 3 years. For removal, the incision should be made parallel to the implant, at the distal extremity of the device, close to the cubital fossa, but this principal was not respected during the initial attempt to remove the implant.^3^

On the basis of the case description, the clinical picture was compatible with a brachial pseudoaneurysm progressing to compression of the nerve, in view of the pain, paresthesia, and restricted ability to extend the arm. If presence of injury to the median nerve is confirmed, the patient should be operated on immediately, to avoid development of permanent neurological sequelae with consequent manual dysfunction.^2^^,^^4^^,^^5^ The preference for local anesthesia and conscious sedation is because of the need to conduct an intraoperative neurological assessment, after decompression of the pseudoaneurysm, and also because of sufficient access to obtain proximal and distal control of the brachial artery in the arm, which would have enabled us to extent the approach if necessary.

In this case, we observed that incorrect implantation of an SCI could cause a partial injury to the left median nerve and that the poorly executed attempt to remove it culminated in arterial injury, formation of a brachial artery pseudoaneurysm, and consequent exacerbation of the nerve damage. Open surgery was therefore chosen to repair the pseudoaneurysm, remove the implant, and evacuate the hematoma, with consequent relief of nerve compression. Compression of the median nerve in the arm is very uncommon and because of its rarity it is sometimes neglected during differential diagnosis.^4^^,^^5^

For many years, the only treatment option was immediate open repair to prevent expansion of the pseudoaneurysm.^6^ Nowadays, there are variations on the same technique and less invasive options. Noninvasive options include conservative treatment with meticulous outpatients monitoring for small pseudoaneurysms and compression with Doppler ultrasound. However, these methods are limited to cases with small diameter, narrow neck, and recent onset.^6^^-^8 The most common minimally invasive techniques are injection of thrombin with or without factor XIII and deployment of covered stents, anatomic location permitting.^6^^-^8 Both require expensive materials that are not always available in all hospitals. In the present case, ultrasound-guided compression or injection of thrombin were contraindicated because of the considerable pain and restriction of movement of the limb, in addition to the nerve damage. Ambulatory procedures to implant or explant SCIs should be performed in a manner compatible with the vascular anatomy of the chosen limb and by professionals with expertise in the procedure used.
