# Supplementary material for: Traumatic pseudoaneurysm in brachial artery after removal of a subdermal contraceptive implant
Source: J Vasc Bras. 2020 Sep 21;19:e20200040. doi: 10.1590/1677-5449.200040 (PMC8217999; doi:10.1590/1677-5449.200040)
Supplement: Translation

# Translation: Pseudoaneurisma traumático em artéria braquial após retirada de implante contraceptivo subdérmico

**Authors:** Rafael Borges Monteiro, Patrick Bastos Metzger, Ariadne Bonachela de Moura, Antonio Herculano Silva, Matheus Nogueira Campos, Arthur Suana de Brito, Mirella Prado Luengo, Maria Júlia Andrade Nascimento

**Affiliations:** 1 Hospital Municipal do Campo Limpo – HMCL, Departamento de Cirurgia Vascular e Endovascular, São Paulo, SP, Brasil.; 2 Hospital Universitário Professor Edgard Santos, Salvador, BA, Brasil.; 3 Centro Universitário das Américas – CAM, Curso de Medicina, São Paulo, SP, Brasil.; 4 Beneficência Portuguesa de São Paulo, Departamento de Cirurgia Vascular, São Paulo, SP, Brasil.; 5 Universidade Anhembi Morumbi – UAM, São Paulo, SP, Brasil.

**Keywords:** lesões do sistema vascular, artéria braquial, anticoncepcionais

## Abstract

Os procedimentos terapêuticos invasivos têm aumentado frequentemente com a evolução da medicina, consequentemente aumentando o número de complicações decorrentes deles. O dispositivo contraceptivo subdérmico (DCS) tem um benefício para a contracepção feminina, mas o implante e a retirada apresentam uma taxa de complicações em torno de 3%. Neste artigo, relatamos e discutimos um caso de pseudoaneurisma traumático da artéria braquial após tentativa de retirada do implante, complicada com a compressão do nervo mediano.

## INTRODUÇÃO

Pseudoaneurismas são uma complicação cada vez mais frequente devido ao grande aumento de procedimentos invasivos, diagnósticos e terapêuticos^1^^,^^2^. O pseudoaneurisma em artéria braquial é uma condição rara e ocorre em menos de 0,04% dos casos^1^. Sua principal etiologia são as causas iatrogênicas (punção ou exames de diagnóstico), estando presente entre 0,3 e 0,7% dos pacientes e, por consequência, aumentando a morbimortalidade, o tempo de hospitalização e os custos para o sistema de saúde^1^^,^^2^.

O dispositivo contraceptivo subdérmico (DCS) vem aumentando sua frequência de utilização para contracepção e reposição hormonal feminina nos últimos 5 anos, apresentando uma taxa de complicação total em torno de 3%^3^. Os autores apresentam um caso clínico incomum de pseudoaneurisma braquial causado pela tentativa de retirada do DCS, complicada com compressão do nervo mediano.

## DESCRIÇÃO DO CASO

A paciente era uma jovem de 18 anos, primípara, admitida no hospital pelo setor de Ginecologia e Obstetrícia para retirada de DCS implantado há 1 mês. O implante havia sido realizado em região medial de braço, tendo evoluído após o procedimento com dor, edema local e equimose. Durante o acompanhamento ambulatorial de revisão, a paciente apresentou parestesia, perda progressiva da força de flexão do dedo e hipestesia no território do nervo mediano esquerdo. A equipe de ginecologia assistente optou, então, pela retirada precoce do dispositivo durante o acompanhamento ambulatorial pós-operatório.

Durante a manipulação cirúrgica inicial, houve relato, da equipe da ginecologia, de grande dificuldade na retirada do contraceptivo, em uma abordagem cirúrgica em face medial do braço na extremidade proximal do implante, em que se optou pela interrupção do procedimento. Ao final, a paciente encontrava-se com importante dor local, restrição de extensão do membro e aumento do volume do membro, com o aparecimento de massa pulsátil, além de parestesia distal. Nesse momento, foi acionada, no mesmo dia, a equipe de cirurgia vascular devido à suspeita de pseudoaneurisma braquial (Figura 1). Ao exame, observou-se massa pulsátil em face medial do terço distal do braço. Além disso, não foi possível palpar o DCS em seu trajeto, por provável volume do hematoma local. O ultrassom Doppler colorido demonstrou a presença de pseudoaneurisma com trombos em sua parede, com diâmetros de 4,2 x 3,5 cm, na face medial de artéria braquial, apresentando colo de 6 mm e fluxo turbilhonar (Figura 2).

**Figura 1:** (**A**) Volumoso hematoma em terço distal de braço esquerdo. Note a incisão medial em terço médio de braço para tentativa de retirada de implante contraceptivo subdérmico; (**B**) Tratamento cirúrgico aberto do pseudoaneurisma de braquial. Retirada total do hematoma, descompressão do nervo e correção do orifício arterial; (**C**) Implante contraceptivo subdérmico retirado.

**Figura 2:** (**A**) Ultrassonografia duplex pré-operatório em corte longitudinal demonstrando a descontinuidade arterial do pseudoaneurisma (A BRAQ = Artéria Braquial); (**B**) Fluxo turbilhonar em “yin yang” no pseudoaneurisma.

Optou-se por intervenção cirúrgica aberta imediata para a correção da lesão, devido ao comprometimento neurológico do membro. O procedimento foi realizado sob anestesia local e leve sedação, com incisão medial do braço e identificação da lesão arterial em artéria braquial e compressão nervosa local. Foi realizada sutura do orifício arterial, assim como evacuação do hematoma local. No pós-operatório imediato, a paciente apresentava-se com pulsos distais amplos e simétricos, perfusão adequada, motricidade e sensibilidade preservadas distalmente.

No seguimento, a paciente evoluiu em 14 dias com total remissão da dor e recuperação plena da capacidade motora com extensão total do antebraço e ausência de déficit de flexão, parestesia ou hipestesia em dedos. Pulsos distais mantendo-se amplos e simétricos bilateralmente.

## DISCUSSÃO

O pseudoaneurisma da artéria braquial é uma complicação rara e geralmente tem origem traumática ou iatrogênica^1^. É mais comumente vista em usuários de drogas injetáveis e em pacientes em hemodiálise^1^^,^^2^. Neste trabalho, relatamos um caso incomum de pseudoaneurisma braquial iatrogênico após a retirada de DCS. Essas complicações podem evoluir para condições como ruptura hemorrágica, compressão dos nervos e veias, formação de edema importante levando a redução da perfusão distal, bem como necrose local da pele devido à isquemia^1^^,^^2^^,^^4^^,^^5^.

Os pseudoaneurismas com 2 cm ou menos tendem a ser assintomáticos. Quando sintomáticos, apresentam-se na maioria das vezes como massa pulsátil. Um quadro de sepse pode estar associado a pseudoaneurismas braquiais quando há infecção associada, decorrente do uso crônico de drogas injetáveis^1^. Os fatores de risco para a formação de pseudoaneurisma incluem *diabetes mellitus* e uma contagem de plaquetas menor que 200.000/ µL. O uso de anticoagulantes e/ou antiagregantes plaquetários pré ou pós-operatórios também constituem risco. Além disso, outros fatores incluem idade avançada, sexo feminino e aumento do índice de massa corporal (IMC)^2^^,^^4^^,^^5^.

O implante ou explante de dispositivo contraceptivo subdérmico pode causar um aumento da frequência das lesões vasculares e neurológicas^3^. A inserção e posterior remoção desse implante estão dentro do conhecimento esperado de um especialista^3^. A maioria dos serviços médicos exige um treinamento, que é oferecido pela fabricante do DCS para que os médicos manejem os implantes e as retiradas. O não cumprimento das instruções de implante pode levar a implantação profunda e impalpável, que oferece risco de migração do implante, lesão neurológica e vascular e, raramente, implantação intravascular^3^. O DCS deve ser retirado ou substituído em 3 anos. Na retirada, a incisão deve ser paralela ao implante na extremidade distal do dispositivo, próximo a prega do cotovelo, preceito este não seguido na tentativa inicial de retirada do implante^3^.

A partir do relato, pode-se suspeitar de uma clínica compatível com um pseudoaneurisma braquial evoluindo para uma compressão neurológica, devido a dor, parestesia e restrição da extensão do membro. A presença de uma lesão no nervo mediano, se confirmada, deve levar o paciente a ser operado imediatamente a fim de evitar que se desenvolvam sequelas neurológicas permanentes com consequente disfunção manual^2^^,^^4^^,^^5^. A preferência pela realização do procedimento com anestesia local e sedação consciente se deve pela necessidade de avaliação neurológica no intraoperatório, após descompressão do pseudoaneurisma, bem como pelo acesso adequado ao controle proximal e distal da artéria braquial no braço, o que nos daria a possibilidade de ampliação do acesso, caso necessário.

Neste relato, observamos que o implante incorreto do DCS pode ter causado uma lesão parcial do nervo mediano esquerdo, e a tentativa inadequada de retirada do implante culminou na lesão arterial e formação do pseudoaneurisma de artéria braquial, com consequente piora neurológica. Portanto, a cirurgia aberta foi a escolhida para o tratamento do pseudoaneurisma, a retirada do implante e a evacuação do hematoma, com consequente diminuição da compressão nervosa. A compressão do nervo mediano no braço é bastante incomum e sua raridade às vezes é negligenciada no diagnóstico diferencial^4^^,^^5^.

A correção aberta imediata para prevenir expansão do pseudoaneurisma foi durante muitos anos a única opção terapêutica^6^. Contemporaneamente, há variações da mesma técnica e alternativas menos invasivas. Entre as táticas não invasivas, temos o tratamento conservador, com o seguimento ambulatorial meticuloso dos pseudoaneurismas pequenos e a compressão com ultrassom Doppler. No entanto, essas últimas ficam restritas a casos com pequeno diâmetro, colo estreito e pouco tempo de evolução^6^^-^8. As técnicas minimamente invasivas mais utilizadas são a injeção de trombina com ou sem fator XIII e o implante de stent recoberto, quando a localização anatômica permitir^6^^-^8. Ambas requerem solicitação de materiais de custo elevado e nem sempre disponíveis em todos os hospitais. Neste caso, foi contraindicada a compressão ecoguiada ou a injeção de trombina devido à importante dor e limitação da movimentação do membro, bem como a presença de lesão neurológica. Procedimentos ambulatoriais para implante ou explante de DCS devem ser realizados consoante a anatomia vascular do membro escolhido e por profissionais com expertise no procedimento proposto.
